# P176: Thinking critically on the issue of hand hygiene: a case study of a clinical seminar, for nursing students, on the subject of infection control and prevention

**DOI:** 10.1186/2047-2994-2-S1-P176

**Published:** 2013-06-20

**Authors:** I Livshiz Riven, N Hurvitz, A Kopitman, JL Reishtein, V Shor, R Nativ

**Affiliations:** 1 Nursing, Ben Gurion of the Negev, Beer-Sheva, Israel; 2 Infection Control Unit, Soroka University Medical Center, Beer-Sheva, Israel; 3 Division of Obstetrics and Gynecology, Soroka University Medical Center, Beer-Sheva, Israel

## Introduction

In the complex world of modern healthcare it is vital that nurses possess good critical thinking skills. Infection control and prevention (IC&P) in healthcare is a high impact issue, and adherence of health care workers to Hand Hygiene (HH) guidelines is a major topic.

## Objectives

Development of nursing students' ability to apply critical thinking skills in a clinical seminar on the subject of IC&P, using the example of adherence to HH guidelines.

## Methods

A 4-credit course on the subject of IC&P was offered to third year baccalaureate nursing students as a clinical seminar. During the first semester students learned to critically evaluate published research and academic writing. During the second semester the students performed a research project, as part of the activities of the IC&P Program in a large tertiary hospital. The students examined the attitudes, knowledge, and practices regarding HH among nurses and nursing aides in the obstetric division. They also wrote a portfolio about their experiences.

## Results

Seventeen students (20% of the class) chose the IC&P clinical seminar. Towards the end of the seminar students prepared a critical analysis of the literature and their findings and explanations. They presented HH compliance rates, and analyzed the nurses' knowledge, attitudes, and beliefs regarding HH. They found a growing awareness of infection control needs and that nurses know that compliance is lower than desirable; some believe this is due to workloads and others that HH isn't always necessary for healthy patients (mothers). All of the students felt that the course contributed to their ability to critically evaluate behaviors and beliefs regarding the HH challenges.

## Conclusion

This course succeeded in making students recognize the need to challenge common health care setting reasoning. It was a successful collaboration between the nursing education system and the healthcare service with awakened awareness to sub-textual information in the context of HH.

## Disclosure of interest

None declared

